# Fibrinogen to Albumin Ratio, Lactate Dehydrogenase to Albumin Ratio and Uric Acid to Albumin Ratio in Preeclampsia

**DOI:** 10.3390/jcm15010001

**Published:** 2025-12-19

**Authors:** Esra Selvi, Kübra Kurt Bilirer, Aybekcan Batman, İzel Günay, Verda Alpay, Hakan Erenel

**Affiliations:** 1 Department of Perinatology, Başakşehir Çam and Sakura City Hospital, 34480 Istanbul, Turkey; kubrakbilirer@gmail.com (K.K.B.); batman.aybek.can@gmail.com (A.B.); verda_alpay@yahoo.com (V.A.); hakanerenel@yahoo.com (H.E.); 2 Department of Obstetrics and Gynecology, Başakşehir Çam and Sakura City Hospital, 34480 Istanbul, Turkey; zel.gunay@gmail.com

**Keywords:** albumin, fibrinogen, LDH, preeclampsia, uric acid

## Abstract

**Background/Objectives**: Preeclampsia can be divided into two groups (with and without severe features) based symptom severity. We aimed to distinguish these two entities with the aid of fibrinogen to albumin ratio (FAR), uric to acid albumin ratio (UAR) and LDH to albumin ratio (LAR). **Methods**: This retrospective study was conducted in Istanbul Basaksehir Cam and Sakura City Hospital between 2020 and 2023. Seventy-three patients with preeclampsia were included in this study which were categorized into two groups according to disease severity: 40 patients with preeclampsia without severe features and 33 patients with severe features. Additionally, 30 healthy pregnant women were included as a control group. Neutrophil–lymphocyte ratio (NLR), monocyte–lymphocyte ratio (MLR), platelet–lymphocyte ratio (PLR), mean platelet volume (MPV), red cell distribution width (RDW), mean platelet volume (MPV), Uric acid, LDH, AST, ALT, fibrinogen, albumin, FAR, UAR and LAR were compared among the groups. **Results**: FAR was significantly higher in preeclampsia patients with and without severe features compared to control group (Odds ratio 8.32 for ≥0.139 vs. <0.139, *p* < 0.001). There was no significant difference in FAR levels between preeclampsia patients according to disease severity. UAR and LAR were significantly different between preeclampsia patients with and without severe features and the control group (*p* < 0.001). Receiver operating characteristics (ROC) curves for UAR showed that a cut-off value of 1.727 had a sensitivity of 73% and a specificity of 68% in discriminating between preeclampsia with and without severe features (Odds ratio 5.53 for ≥1.727 vs. <1.727). ROC curves for LAR showed that a cut-off value of 79.09 had a sensitivity of 85% and a specificity of 73% in discriminating between preeclampsia with and without severe features (Odds ratio 14.76 for ≥79.09 vs. <79.09). **Conclusions**: UAR and LAR appear to be better markers than FAR for identifying preeclamptic patients who require delivery due to severe features. They are easily accessible and promising biomarkers, and to our knowledge, this is the first study to evaluate LAR in this context. Further studies are needed to validate their diagnostic accuracy and compare their performance with established biomarkers.

## 1. Introduction

Preeclampsia is one of the leading causes of maternal and neonatal morbidity and mortality, occurring in 5–10% of pregnancies and characterized by newly developed hypertension and proteinuria after 20 weeks of gestation [1,2]. Although endothelial cell activation, intravascular inflammation, and syncytiotrophoblast stress play crucial roles in the underlying pathophysiology, the exact pathological mechanism of preeclampsia remains unclear [3]. Adequate trophoblastic invasion of the spiral arteries and low-resistance flow in these vessels are essential for a normal pregnancy. In contrast, shallow trophoblastic invasion and high-resistance flow are characteristic features of preeclampsia pathogenesis. The involvement of immune and pro-inflammatory cells is crucial for the proper regulation of trophoblastic invasion. An increase in pro-inflammatory cells, combined with a decrease in regulatory immune cells, leads to an imbalance that causes a chronic inflammatory state, promoting the pathogenesis of preeclampsia [4]. Consequently, previous studies have investigated systemic inflammatory markers, such as the neutrophil-to-lymphocyte ratio (NLR), monocyte-to-lymphocyte ratio (MLR), and platelet-to-lymphocyte ratio (PLR), to differentiate preeclampsia with severe features (sPE) from preeclampsia without severe features [5,6]. These markers were also evaluated in comparisons between preeclamptic and normotensive pregnant women [7]. The studies demonstrated the usefulness of these markers in diagnosing preeclampsia and differentiating its severity [8]. Wang et al. found that NLR is a better marker than MLR in differentiating disease severity, and a cutoff value of NLR > 4.182 demonstrated optimal performance with a sensitivity of 69.68%, specificity of 63.95%, and an AUC of 0.71 [5]. However, another study reported better performance for PLR compared to NLR and MLR in differentiating disease severity [6]. Coagulation and platelet activation at the maternal–fetal interface are also implicated in the development of preeclampsia [9]. Mean platelet volume (MPV) and red blood cell (RBC) distribution width (RDW) are indirect markers of platelet reactivity, thrombotic disorders, and cardiovascular disease [10,11]. Accordingly, MPV and RDW have previously been evaluated in preeclampsia [6,12]. Yilmaz et al. showed that mean RDW values were higher in sPE compared to preeclampsia without severe features [12]. A systematic review and meta-analysis showed that MPV is a helpful marker for the diagnosis and follow-up of preeclampsia [13]. All of these markers share the advantage of being inexpensive and easily obtainable in all preeclamptic patients.

Uric acid is one of the most intensely studied laboratory markers in gestational hypertension and preeclampsia. It has been shown that elevated uric acid levels are a common finding in preeclampsia due to decreased glomerular filtration, enhanced tubular reabsorption, and diminished tubular secretion [14]. Uric acid is a clinically useful marker for differentiating preeclampsia from gestational hypertension and sPE from without severe features [15,16]. Lactate dehydrogenase (LDH) is an enzyme found in almost all tissues, but mainly in the muscle, liver, kidney, and red blood cells [17]. LDH is a marker of hemolysis and is essential for diagnosing HELLP syndrome; however, hemolysis is not a diagnostic criterion for sPE. Nevertheless, previous studies have shown that LDH levels are associated with disease severity [18,19]. A recent study evaluated the role of the fibrinogen-to-albumin ratio (FAR) as a novel inflammatory marker in preeclampsia and found that FAR is a useful marker for predicting disease severity [20]. Although the uric acid-to-albumin ratio (UAR) has previously been evaluated in preeclampsia in relation to disease severity, the LDH-to-albumin ratio (LAR) has not been reported [21,22].

sPE tends to develop preterm and is often accompanied by severe hypertension, more pronounced clinical manifestations—such as persistent headache, severe epigastric or right upper quadrant pain, and various visual disturbances—as well as laboratory abnormalities, including elevated AST and ALT levels and increased creatinine, indicating significant end-organ involvement [23]. However, it is not always possible to distinguish between the two different entities of preeclampsia, as symptoms such as headache and visual disturbances may be misleading, and hypertension may not be persistent. In our clinical practice, the majority of patients with suspected sPE have been hospitalized. While some patients with severe hypertension respond to antihypertensive therapy or experience symptom relief and can deliver at term without developing severe features, others continue to exhibit persistent severe features and require delivery due to sPE. In this study, we aimed to evaluate FAR, UAR, LAR, and other inflammatory markers, including NLR, MLR, RDW, and MPV, in distinguishing sPE from those without severe features.

## 2. Materials and Methods

### 2.1. Study Design and Ethical Approval

This retrospective study was conducted at Istanbul Basaksehir Cam and Sakura City Hospital. After approval by the local ethics committee (approval date and number: 6 November 2023; KAEK/25.10.2023.521), seventy-three patients with preeclampsia who were followed in our perinatology department and delivered at our hospital between August 2020 and October 2023 were included in this study. Seventy-three patients with preeclampsia were categorized into two groups according to disease severity: 40 patients with preeclampsia without severe features and 33 patients with sPE. Additionally, 30 healthy pregnant women were included as a control group.

### 2.2. Definitions and Etiological Classifications

Preeclampsia was diagnosed based on the following criteria: new-onset hypertension (systolic blood pressure ≥ 140 mmHg and/or diastolic blood pressure ≥ 90 mmHg measured on two occasions separated by at least 4 h) at or after 20 weeks of gestation, and proteinuria (≥300 mg in 24 h) [23]. Patients were categorized by symptom severity. A diagnosis of sPE was made with one or more of the following criteria: systolic blood pressure of 160 mmHg or more, or diastolic blood pressure of 110 mmHg or more on two occasions at least 4 h apart, low platelet count (less than 100 × 10^9^/L), increased serum liver enzyme levels (exceeding twice the upper limit of normal), or by severe persistent right upper quadrant or epigastric pain unresponsive to medications, renal insufficiency (serum creatinine concentration > 1.1 mg/dL, or a twofold increase without underlying renal disease), pulmonary edema, newly developed headache resistant to medication, visual disturbances [23].

### 2.3. Participants

Eligible patients were identified using our institution’s electronic medical records system. Patients who were hospitalized due to suspected sPE and subsequently discharged (either because their symptoms resolved or their hypertension was controlled with antihypertensive therapy) were followed in the outpatient clinic and classified as having preeclampsia without severe features.

The control group was selected from patients who attended our hospital for routine pregnancy care. Exclusion criteria for both the control and preeclampsia groups were as follows: chronic hypertension, gestational hypertension, pregestational diabetes mellitus, rheumatologic disorders, acute or chronic infections, cardiac diseases, placenta previa, cholestasis of pregnancy, preterm delivery, HELLP syndrome, abnormal liver enzyme levels, hematologic disorders, and multiple pregnancies. Gestational age was assessed based on the first day of the last menstrual period and confirmed by first-trimester crown-rump length measurement.

### 2.4. Data Collection and Variables

Data on preeclamptic patients were extracted from the perinatology department and emergency room records. In our routine, venous blood samples were collected from patients after the diagnosis of preeclampsia and before any medication was administered. The control group was selected from patients in the routine antenatal care database. Complete blood count (CBC) parameters (white blood cell, neutrophil, monocyte, lymphocyte, and platelet counts, MPV, and RDW); serum urea, creatinine, transaminases (AST and ALT), lactate dehydrogenase (LDH), uric acid, fibrinogen, and albumin levels were extracted from the database. FAR, UAR, LAR, NLR, MLR, and PLR were calculated. Laboratory parameters were compared among the preeclampsia groups and the control group.

Neutrophil, lymphocyte, monocyte, platelet, MPV, and RDW were measured with impedance and fluorescent flow cytometry using the Sysmex XN-1000 (Sysmex, Kobe, Japan) analyzer. LDH, uric acid, and albumin were measured on the Roche Cobas 8000 analyzer (Roche Diagnostics, Basel, Switzerland) in our institutional central laboratory. Fibrinogen analysis was performed on the Cobas t-711 coagulation analyzer (Roche Diagnostics, Mannheim, Germany) in our institutional central laboratory.

### 2.5. Statistical Analysis

All statistical analyses were performed using SPSS version 20 for Windows (SPSS, Inc., Chicago, IL, USA). Data are expressed as mean, standard deviation, median, minimum value, and maximum value. Before comparing the variables, the distributions of the continuous variables were assessed for normality by performing the Kolmogorov–Smirnov and Shapiro–Wilk tests. Multiple group comparisons were conducted using 1-way analysis of variance (ANOVA) or the Kruskal–Wallis test. For group-to-group comparisons, the Bonferroni correction was used to identify which groups differed significantly. In the preeclampsia group, receiver operating characteristic (ROC) curves were generated, and the area under the curve (AUC) was calculated for each marker. Sensitivity and specificity were calculated using the cut-off points determined from the ROC curves. According to the cutoff value obtained from the Youden index, unadjusted (crude) odds ratios were calculated using logistic regression. A *p*-value of <0.05 was considered statistically significant.

## 3. Results

The demographic and clinical characteristics of preeclamptic patients and healthy controls are shown in Table 1. The BMI of preeclampsia patients without severe features was significantly higher than that of the control group and sPE groups (*p* < 0.001). Gestational age at preeclampsia diagnosis and at the time of blood sampling were similar across groups (*p* > 0.05). Mean gestational age at delivery was 39, 36.5, and 33.2 weeks in the control, preeclampsia without severe features, and sPE groups, respectively. There was a clinically significant difference between the groups in gestational age at delivery (*p* < 0.001).

The patients’ laboratory parameters are presented in Table 2. There were no statistically significant differences in the NLR, MLR, PLR, and RDW between the groups. The mean MPV values were significantly higher in the sPE group (*p* = 0.021); however, there was no difference between preeclampsia patients according to severity (*p* > 0.05). The mean uric acid levels were 3.7, 5.3, and 6.3 mg/dL in the control, preeclampsia without severe features, and sPE groups, respectively (*p* < 0.001). The mean LDH levels were 186, 244 and 328 U/L in the control, preeclampsia without severe features, and sPE groups, respectively (*p* < 0.001). The uric acid and LDH levels were significantly different between the groups. Fibrinogen levels were markedly higher in the patients without severe features than in the control group (*p* = 0.012). Albumin levels were significantly lower in the sPE group compared to both the control group and preeclampsia patients without severe features (*p* < 0.001). The FAR was considerably higher in preeclampsia patients with and without severe features than in the control group (Odds ratio 8.32 for FAR ≥ 0.139 vs. <0.139, 95% CI: 2.63–26.29; *p* < 0.001). However, there was no difference between preeclampsia patients according to severity (*p* > 0.05). UAR and LAR were significantly different between preeclampsia patients with and without severe features and the control group (*p* < 0.001).

**Figure 1:**
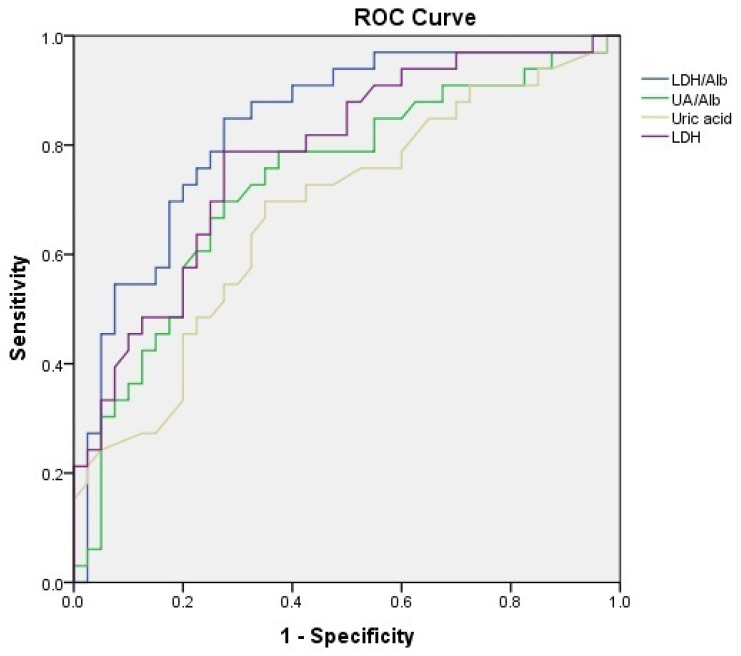
ROC curves illustrating the discriminatory performance of LDH, uric acid, UAR, and LAR in differentiating preeclampsia with and without severe features. features.

The ROC curve analysis was used to further clarify the diagnostic value of these parameters (Figure 1). ROC curves for LDH showed that a cut-off value of 258.5 U/L had a sensitivity of 78% and a specificity of 72% in discriminating between preeclampsia with and without severe features. ROC curves for uric acid showed that a cut-off value of 5.65 mg/dL had a sensitivity of 70% and a specificity of 65% in discriminating between preeclampsia with and without severe features. ROC curves for UAR showed that a cut-off value of 1.727 had a sensitivity of 73% and a specificity of 68% in discriminating between preeclampsia with and without severe features (Odds ratio 5.53, 95% CI: 2.01–15.24; *p* = 0.001). ROC curves for LAR showed that a cut-off value of 79.09 had a sensitivity of 85% and a specificity of 73% in discriminating between preeclampsia with and without severe features (Odds ratio 14.76, 95% CI: 4.54–47.94; *p* < 0.001). The results of the ROC analysis of LDH, uric acid, UAR, and LAR for discriminating between preeclampsia with and without severe features are shown in Table 3.

## 4. Discussion

Preeclampsia is a pregnancy-related multisystem disorder that is a leading cause of maternal mortality and morbidity [1]. Obesity increases the risk of developing preeclampsia; nevertheless, evidence regarding whether higher BMI is associated with more severe forms of the disease is inconclusive [24]. Since BMI values were higher in the non-severe group compared with the sPE group, this may suggest that placental factors play a more dominant role in determining disease severity. Moreover, it has been demonstrated that higher BMI is more strongly associated with non-severe forms of preeclampsia than with sPE [25]. In accordance with previous literature, we found that the gestational age at delivery was earlier in patients with severe features compared to those without severe features [26].

Recent studies focused on the early prediction of preeclampsia and the reduction in adverse outcomes in preeclampsia [27]. However, distinguishing between preeclampsia with and without severe features is also critical for disease management. In clinical practice, this is not always straightforward. Numerous serum parameters have been reported in the literature as potential markers to differentiate sPE from preeclampsia without severe features. A recent study showed that FAR and UAR have predictive value for the severity of preeclampsia [20,22]. We aimed to investigate this novel marker, as well as other known markers (NLR, MLR, PLR, MPV, RDW), and LAR in our population. Although FAR was higher in the preeclampsia group, it was not effective in discriminating between preeclampsia with and without severe features. Ren et al. defined severe proteinuria (>3 + on dipstick or proteinuria > 5 g/24 h urine collection) as a criterion for severe preeclampsia; however, severe proteinuria is neither a diagnostic criterion for sPE nor an indication for delivery with any hypertensive disorder of pregnancy according to the new guidelines for gestational hypertension and preeclampsia [20,23,28]. Since severe proteinuria was not included as a diagnostic criterion for sPE in our study, the comparable FAR results in both preeclampsia groups are easily understandable. Although we could not demonstrate higher fibrinogen levels in sPE than in those without severe features, Ren et al. showed higher fibrinogen levels in sPE [20]. In contrast to this study, Chen et al. demonstrated that fibrinogen levels were significantly decreased in sPE [29]. They also defined proteinuria (>5 g protein in a 24 h urine sample) as a criterion for severe features. The opposite results for fibrinogen levels in sPE may be explained by differences in inclusion criteria and definitions of sPE across studies.

In accordance with previous studies, we found higher uric acid levels in the preeclampsia group. The sPE group had significantly higher uric acid levels than the preeclampsia group without severe features. Sudjai et al. evaluated uric acid levels in patients with preeclampsia and found mean values of 6.44 ± 1.44 mg/dL in the severe group and 5.87 ± 1.53 mg/dL in the non-severe group, similar to our findings [16]. ROC analysis was not performed for preeclampsia; however, they reported that uric acid levels higher than 7 mg/dL were associated with an odds ratio of 2.56 for severe preeclampsia [16]. In our study, we observed that UAR demonstrated better sensitivity and specificity than uric acid alone in distinguishing sPE from preeclampsia without severe features. UAR was evaluated as a predictor of sPE in a previous study, which found no difference in UAR between mild and severe preeclampsia [21]. Their results may have been statistically insignificant because of the small sample size. A recent study by Dayanan et al. evaluated the UAR, FAR, and uric acid-to-creatinine ratio in preeclamptic patients and reported that UAR had the highest accuracy for differentiating disease severity (AUC: 0.708) [22]. In our study, UAR demonstrated superior diagnostic performance, yielding an AUC of 0.731. Dayanan et al. suggested that their differing UAR results compared with the previous study may be due to differences in blood sampling timing and the inclusion of patients with chronic hypertension, as these factors may influence baseline uric acid levels. Our findings were similar to those reported by Dayanan et al., which may be explained by the comparable methodology and the larger sample size, in contrast to the first study on UAR in preeclampsia by Mohamed et al. Our study is the third to evaluate UAR in relation to preeclampsia severity, and further studies are needed to confirm our findings.

Preeclampsia is a significant risk factor for HELLP syndrome, a multisystemic disorder presenting with hemolysis, elevated liver enzymes, and low platelet count syndrome [30]. LDH levels, platelet count, and liver enzyme levels are essential in preeclamptic patients for excluding HELLP syndrome. In our study, we found that LDH levels were significantly different among the three groups. Jaiswar et al. showed a correlation between LDH and the severity of preeclampsia [31]. In their study, the mean LDH level was 646 IU/L in the sPE group, which is clearly higher than our results. Burwick et al. evaluated LDH levels in relation to preeclampsia severity and reported a median LDH level of 215 IU/L in patients with sPE [32]. In our study, the mean LDH level was 328 IU/L in the sPE group. Variations in LDH assays, gestational age at the time of serum sampling, and different criteria for sPE may explain differences in LDH levels. We found that the LAR provided better sensitivity and specificity than LDH alone (AUC: 0.708 vs. 0.832) in distinguishing preeclampsia with and without severe features. LAR was the most discriminatory marker in our study for distinguishing preeclampsia severity. The enhanced capability of this marker compared with LDH alone may be explained by the association between decreased albumin levels and disease severity [33]. In accordance with this, albumin levels were also significantly lower in the sPE group than in patients without severe features.

In our study, inflammatory markers, including NLR, MLR, PLR, and RDW, did not differ significantly between groups. Meta-analyses have demonstrated the roles of NLR and RDW in the clinical prediction and severity assessment of preeclampsia [8,34]. A recent study evaluated NLR in 84 patients with non-severe and 34 with severe preeclampsia and found no significant difference between the groups [6]. The contradictory results reported in the literature may be explained by differences in the timing of blood sampling, the diagnostic criteria used, the gestational age at sampling, and the sample size. Although we observed a trend of decreasing PLR with increasing disease severity, the median levels did not differ significantly among the three groups. Kim et al. evaluated PLR in women with preeclampsia and found that PLR was significantly lower in the severe preeclampsia group compared with the non-severe group [35]. This study included 227 patients with severe preeclampsia, representing a much larger sample size than ours. In our study, LAR demonstrated superior performance compared to inflammatory markers in distinguishing disease severity.

The imbalance between angiogenic growth factors such as placental growth factor (PlGF) and vascular endothelial growth factor (VEGF) and the antiangiogenic factor soluble fms-like tyrosine kinase-1 (sFlt-1) plays a central role in the pathophysiological mechanisms of preeclampsia [36]. The value of angiogenic factors in predicting and diagnosing preeclampsia has been demonstrated in previous studies [36,37]. Moreover, some international guidelines (NICE, Swiss Society for Obstetrics and Gynecology, and German Society of Obstetrics and Gynecology guidelines) now recommend the use of the maternal sFlt-1/PlGF ratio for both the diagnosis and prediction of preeclampsia [36]. The principle behind using angiogenic factors is to evaluate patients with suspected preeclampsia, help rule out the disease, and reduce unnecessary hospitalizations. However, we aimed to assess laboratory parameters in patients with confirmed preeclampsia. A major disadvantage of using angiogenic factor testing is its limited worldwide availability and high cost. However, LDH, uric acid, UAR, and LAR can be easily evaluated in all patients with preeclampsia. Especially, LAR appears to be a promising marker for distinguishing sPE from preeclampsia without severe features.

This study had several limitations, including its retrospective nature, relatively limited sample size, the absence of neonatal outcome data, and the lack of assessment of associations between parameters and postnatal outcomes.

## 5. Conclusions

Preeclampsia management constitutes a major proportion of the clinical workload in a tertiary maternal–fetal medicine center. It is not always straightforward to predict which patients with preeclampsia will develop severe features. LAR and UAR are easily accessible and promising markers for identifying preeclamptic patients who require delivery due to severe features. To our knowledge, this study is the first to evaluate LAR in the literature. Further studies are warranted to confirm its diagnostic accuracy and to compare its performance with other established biomarkers.

## Figures and Tables

**Table 1:** Demographic and clinical characteristics of the patients.

	Control Group (*n* = 30)	Preeclampsia Without Severe Features (*n* = 40)	sPE (*n* = 33)	*p* Values	Post Hoc Comparisons *p* Values		
					Control-mPE	Control-sPE	mPE-sPE
Maternal age (years)	29.3 ± 6.38	29.8 ± 6.36	30.5 ± 6.56	NS			
BMI (kg/m^2^)	30.02 ± 4.26	35.13 ± 7.05	29.91 ± 5.67	<0.001	0.003	0.999	0.002
Gravida (n)	2 (1–6)	2 (1–8)	2 (1–7)	NS			
Parity (n)	1 (0–4)	2 (0–7)	1 (1–6)	0.067			
GA at diagnosis and blood sampling (week)	30.6 ± 4.93	32.8 ± 3.76	30.3 ± 5.39	NS			
GA at delivery (week)	39.07 ± 1.02	36.59 ± 1.51	33.2 ± 3.71	<0.001	0.001	<0.001	<0.001

BMI, body mass index; GA, gestational age; mPE: preeclampsia without severe features; NS, not statistically significant; sPE: preeclampsia with severe features.

**Table 2:** Laboratory parameters of the patients.

	Control Group (*n* = 30)	Preeclampsia Without Severe Features (*n* = 40)	sPE (*n* = 33)	*p* Values	Post Hoc Comparisons *p* Values		
					Control- mPE	Control- sPE	mPE-spE
NLR	3.56 (2.13–6.93)	3.11 (1.12–10.66)	3.55 (1.41–28.78)	0.289			
MLR	0.35 (0.19–0.61)	0.29 (0.06–0.81)	0.30 (0.06–0.71)	0.394			
PLR	122.6 (72.8–267.6)	111.8 (44.9–200)	85.4 (26.9–483.3)	0.059			
RDW (%)	13.2 (12.1–19.8)	13.7 (12.1–30.6)	13.7 (11.8–24.9)	0.610			
MPV (fL)	11 ± 0.83	11.64 ± 1.26	11.8 ± 1.26	0.021	0.08	0.026	0.999
Uric acid (mg/dL)	3.77 ± 0.74	5.34 ± 1.33	6.36 ± 1.72	<0.001	<0.001	<0.001	0.005
LDH (U/L)	186 ± 33.5	244 ± 60	328 ± 96	<0.001	0.002	<0.001	<0.001
Fibrinogen (g/L)	4.5 ± 0.72	5.37 ± 1.39	5 ± 1.34	0.015	0.012	0.324	0.6
Albumin (g/L)	36.83 ± 1.76	35.32 ± 5.68	31.09 ± 3.97	<0.001	0.326	<0.001	<0.001
FAR	0.12 ± 0.02	0.15 ± 0.06	0.16 ± 0.04	<0.001	0.003	<0.001	0.999
UAR	1.02 ± 0.21	1.60 ± 0.68	2.12 ± 0.78	<0.001	0.001	<0.001	0.002
LAR	50.6 ± 9.19	72.69 ± 33.61	107.82 ± 34.65	<0.001	<0.001	<0.001	<0.001

LDH, lactate dehydrogenase; NLR, neutrophil-to-lymphocyte ratio; MLR, monocyte-to-lymphocyte ratio; PLR, platelet-to-lymphocyte ratio; RDW, red cell distribution width; MPV, mean platelet volume; UA, uric acid; Alb, albumin; FAR, fibrinogen-to-albumin ratio; mPE: preeclampsia without severe features; sPE: preeclampsia with severe features; LAR, LDH-to-albumin ratio; UAR, uric acid-to-albumin ratio. Reference ranges: RDW 12.4–15.1%; MPV 9.1–11.9 fL; uric acid 2.4–5.7 mg/dL; LDH <250 U/L; fibrinogen 1.93–4.12 g/L; albumin 35–52 g/L.

**Table 3:** The results of ROC analysis of LDH, uric acid, UAR and LAR for discriminating between preeclampsia with and without severe features.

	Cut-Off Value	Sensitivity	Specificity	AUC	*p* Value	95% CI
LDH	258.5	78%	72.5%	0.781	<0.001	0.67–0.88
Uric acid	5.65	70%	65%	0.677	0.01	0.55–0.80
UAR	1.727	73%	68%	0.731	0.01	0.61–0.85
LAR	79.09	85%	73%	0.832	<0.001	0.74–0.93

AUC: area under the curve; LDH, lactate dehydrogenase; CI, confidence interval; LAR, LDH-to-albumin ratio; UAR, uric acid-to-albumin ratio.

## Data Availability

The data that support the findings of this study are available upon reasonable request from the corresponding author.
